# Real‐World Outcomes of Prophylaxis With rIX‐FP in Germany: A Prospective, Non‐Interventional Study in Haemophilia B

**DOI:** 10.1111/hae.70319

**Published:** 2026-07-07

**Authors:** Johannes Oldenburg, Swee Wenning, Susanne Holzhauer, Wolfgang Miesbach, Robert Klamroth, Thomas Linhoff, Patrick Sommerer, Christian Pfrepper, Martin Olivieri, all study investigators

**Affiliations:** ^1^ Institute of Experimental Hematology and Transfusion Medicine University Hospital Bonn, Medical Faculty Bonn Germany; ^2^ SRH Kurpfalzkrankenhaus Heidelberg Germany; ^3^ Department of Pediatric Hematology and Oncology Charité University Medicine Berlin Germany; ^4^ University Hospital Frankfurt Frankfurt Germany; ^5^ Department of Haemophilia and Haemostasis Vivantes Hospital Friedrichshain Berlin Germany; ^6^ CSL Behring Hattersheim Germany; ^7^ Division of Hemostaseology University of Leipzig Medical Center Leipzig Germany; ^8^ Paediatric Thrombosis and Haemostasis Unit, Paediatric Haemophilia Centre Dr. Von Hauner Children's Hospital, LMU Munich Munich Germany

**Keywords:** factor IX, Germany, haemophilia B, prophylaxis, treatment outcome

## Abstract

**Introduction:**

Efficacy and safety of albutrepenonacog alfa (rIX‐FP) in people with haemophilia B (PwHB) has been previously demonstrated in clinical trials. However, real‐world data insights are required.

**Aim:**

To describe real‐world effectiveness and tolerability of rIX‐FP in PwHB in Germany.

**Methods:**

Prospective, non‐interventional study conducted across 19 sites in Germany. Patients of any age treated with rIX‐FP were enrolled between March 2018 and February 2024. Patients were monitored every 3–12 months and followed‐up for 3 years or until ≥100 exposure days.

**Results:**

To our knowledge, this is the largest study of factor replacement therapy in PwHB in Germany to date. Of the patients who completed the study (63/73), most (80.8%) had moderate or severe haemophilia B. The mean (standard deviation) duration of observation was 30.5 (8.32) months. For any prophylaxis regimen, the median (range) annualised bleeding rate (ABR) was 0.89 (0.0–9.6) and annualised spontaneous bleeding rate (AsBR) was 0.08 (0.0–5.2). The median ABR was 1.12, 0.68 and 0.13, and the median AsBR was 0.17, 0.00 and 0.06 for those treated with a 7‐, 10‐ or 14‐day regimen, respectively. Investigators rated the overall haemostatic effectiveness as ‘good’ or ‘excellent’ in 94% of cases (based on the worst assessment). Overall, 82 adverse events in 34 patients were reported, none were considered related to rIX‐FP.

**Conclusion:**

This multicentre, non‐interventional study in Germany demonstrates that rIX‐FP prophylaxis is effective and well tolerated in PwHB of all ages in routine clinical practice and reinforces the results of the clinical trials.

**Clinical trial registration:**

The study has been registered in the non‐interventional studies registry at the Paul Ehrlich Institute (German federal authority for vaccines and biomedicines; study code CSL654_5005; NIS number 410).

## 1 Introduction

Haemophilia B (OMIM 306900) is a rare congenital bleeding disorder characterised by a deficiency in or lack of factor IX (FIX) activity [1]. People with severe haemophilia (defined as <1 IU/dL or <1% normal FIX levels in the blood) commonly experience bleeds in the joints, muscles and internal organs [1].

Prophylaxis with FIX concentrates is the current standard of care for people with severe haemophilia B [1]. In severe haemophilia, the objective for prophylaxis is to revert to moderate or mild haemophilia by maintaining factor levels above 3%–5% at all times [1]. Due to their half‐life of ∼18–24 h, standard half‐life (SHL) FIX products require frequent intravenous infusions (2–3 times a week), resulting in a substantial treatment burden on people with haemophilia B (PwHB) and caregivers, and leading to treatment adherence often being reduced [1, 2]. Extended half‐life (EHL) FIX products offer reduced administration frequency, by allowing for higher trough levels, thus potentially improving patient health‐related quality of life and treatment adherence [1, 2].

rIX‐FP (albutrepenonacog alfa, IDELVION, CSL Behring) is an EHL fusion protein containing recombinant FIX (rFIX) fused to human recombinant albumin [3, 4]. As albumin is protected from degradation by pH‐dependent binding to the neonatal Fc‐receptor, rIX‐FP has an increased half‐life compared with rFIX. In a global Phase 3 study in adult/adolescent patients, the mean half‐life of rIX‐FP was 102 h, which was 4.3 times longer than that of prior FIX therapy [5]. The efficacy and safety of rIX‐FP in PwHB has been previously demonstrated in clinical trials. The PROLONG‐9FP clinical trial programme demonstrated effective haemostasis, and a strong safety and tolerability profile, in both adult/adolescent and paediatric patients with moderate‐to‐severe haemophilia B [5, 6, 7, 8, 9]. A Phase 3b extension study of the PROLONG‐9FP programme that followed PwHB for up to 4 years showed that rIX‐FP had a favourable safety profile and was effective at dosing intervals up to 21 days in selected adult patients [8]. Treatment with rIX‐FP therefore allows select PwHB who are well controlled, to receive prophylaxis at intervals of 7, 10, 14 or 21 days [8]. This flexibility may improve both adherence to prophylaxis and quality of life by further reducing treatment burden.

To evaluate the real‐world use of rIX‐FP, data from routine clinical practice are required. Two post‐marketing studies showed that the use of rIX‐FP prophylaxis is associated with improved bleed protection and reduced FIX consumption versus prophylaxis with a prior SHL FIX [10, 11]. In addition to these post‐marketing studies, prospective, multicentre studies are also ongoing to obtain data on the effectiveness and tolerability of rIX‐FP when used to treat PwHB during routine clinical practice.

This prospective, non‐interventional study was conducted to examine the use of rIX‐FP in the real world. The objective was to describe real‐world effectiveness and tolerability data on the use of rIX‐FP in PwHB in Germany.

## 2 Materials and Methods

### 2.1 Study Population

A prospective, multicentre, non‐interventional study was conducted across 19 sites in Germany. Patients of any age, with haemophilia B being treated with rIX‐FP, either on demand or prophylactically, were enrolled between March 2018 and February 2024. Patients with inhibitors and multi‐factor deficiency at enrolment were excluded. Use of rIX‐FP was in‐line with local practice, according to the German Prescribing Information, and at the investigators’ discretion. Patients were routinely monitored every 3–12 months and followed‐up for 3 years or at least 100 exposure days.

### 2.2 Study Endpoints

The primary outcome measures were annualised bleeding rate (ABR) and annualised spontaneous bleeding rate (AsBR). The secondary outcome measures included frequency and dosage of infusions for prophylaxis and the treatment of bleeds (during prophylaxis and on‐demand treatment periods), which included the frequency and dosage of infusions per bleed. Safety outcome measures included the number of adverse events (AEs) and serious adverse events (SAEs), as well as inhibitor development. In addition, patient‐reported outcomes and health‐related quality of life data were collected using responses to health‐related quality of life and patient satisfaction questionnaires (Haemo‐QoL and Haem‐A‐QoL). FIX consumption and absences from work/school due to bleeds were also recorded to evaluate health economic aspects. Other data collected included joint status (arthropathy/target joints) and surgical interventions (including dental). Due to the non‐interventional nature of the study, some endpoints lacked robust data collection and are therefore not included in the results.

### 2.3 Data Collection

Study‐related data were recorded in the electronic case report form (eCRF) by authorised study personnel. Information on infusions, bleeds and surgeries recorded in the patients’ diaries were transferred to the eCRF. Data were collected at baseline, follow‐up visits (every 3–12 months) and at the end of the observation period. Medical history, AEs and surgical procedures during the study were coded using the Medical Dictionary for Regulatory Activities (MedDRA version 21.1). FIX treatment history as well as prior medications and concomitant medications were coded using the WHO‐DD (WHODRUGGlobal Mar 2018 B3 format). All AEs documented in the Electronic Data Capture system were included in the analysis (irrespective of the date of onset, the type of AE and the relationship to rIX‐FP). Adverse drug reactions were defined as AEs causally related to rIX‐FP based on the investigators’ assessment.

### 2.4 Statistical Analysis

Descriptive statistics (mean, standard deviation [SD], median, range and interquartile range) were used to analyse the study results. ABR and AsBR were calculated for each patient depending on the corresponding treatment regimen and summarised separately for prophylaxis and on‐demand treatment. Qualitative/categorical variables were summarised by frequency counts and percentages and, unless otherwise specified, percentages included any missing observations. Data transformations and statistical analyses were performed in SAS version 9.4. The full analysis set included all patients who provided written informed consent and was used for the calculation of baseline characteristics and safety data. The effectiveness data set (EFS) only included patients with an observation period of at least 12 weeks and was used to calculate the primary and secondary endpoints of the study.

### 2.5 Ethics

Written and informed consent was provided by patients and/or a parent/legal guardian in the case of minors. Data management and statistical analyses were carried out by the contract research organisation AMS Advanced Medical Services in coordination with CSL Behring. Patient data were collected, stored and pseudonymised in accordance with General Data Protection Regulations​. This non‐interventional study was conducted in accordance with the requirements and ethical principles set forth in the Declaration of Helsinki, and followed local guidelines for observational studies in Germany using human data. The relevant study documents were submitted to an independent, interdisciplinary ethics committee of the University of Bonn, Germany, prior to the enrolment of any patients. Each participating physician may have also notified their responsible ethics committee according to the ‘Berufsordnung für Ärzte’.

## 3 Results

### 3.1 Study Population

Of the 73 patients who were enrolled in the study between March 2018 and February 2024 (Figure 1), 63 completed the study. The reasons for early discontinuation included lost to follow‐up (*n* = 7), switched to another FIX product (*n* = 2) and other reasons (*n* = 1). One patient did not have final documentation. Patients had a median (minimum [min], maximum [max]) age of 27.0 (1, 80) years and the majority (80.8%) had moderate or severe haemophilia B (*n* = 59) (Table 1). The mean (SD) duration of observation was 30.5 (8.32) months. Most patients (86.3%, *n* = 63/73) had previously received FIX replacement therapy, other than rIX‐FP, at any point prior to the study; the remaining 12.3% (*n* = 9/73) were previously untreated patients (PUPs). Previous FIX treatment data were missing for one patient (1.4%). Other factor concentrates used in the past 12 months (except rIX‐FP) included rFIX (28.6%; nonacog alfa, BeneFIX, Pfizer), rFIX (28.6%; coagulation FIX, Berinin, CSL Behring) and pdFIX (19.0%; human plasma‐derived coagulation FIX, Haemonine, Biotest) (Table 1). rIX‐FP had been received by 87.7% (*n* = 64/73) of patients prior to study enrolment; 36 of these patients had received rIX‐FP within the past 12 months.

**FIGURE 1:**
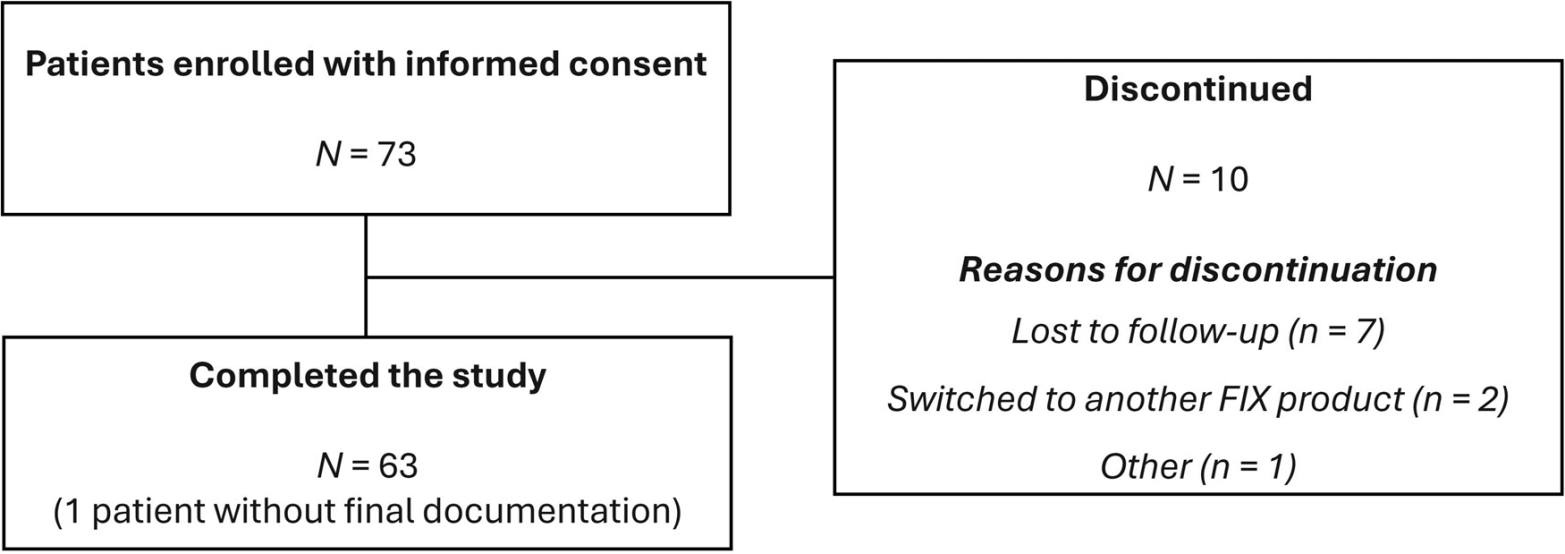
Study design. FIX, factor IX; *N*, total number of patients analysed per group; *n*, number of patients.

**TABLE 1:** Study population characteristics.

	Children <12 years (*n* = 21)	Adolescents 12 – <18 years (*n * = 6)	Adults ≥18 years (*n* = 46)	Total *N* = 73
Sex, *n* (%)				
Male	21 (100)	6 (100)	45 (97.8)	72 (98.6)
Female	0 (0)	0 (0)	1 (2.2)	1 (1.4)
Age				
Mean (SD)	5.2 (3.41)	14.3 (1.75)	39.3 (14.49)	27.4 (19.6)
Median (range)	4.0 (1–11)	14.5 (12–17)	35.0 (18–80)	27.0 (1–80)
Weight (kg)				
Median (range)	18.0 (8–61)	61.2 (37–109)	86.0 (60–134)	75.0 (8–134)
Severity of haemophilia, *n* (%)				
Mild (>5% normal FIX activity)	1 (4.8)	0 (0)	4 (8.7)	5 (6.8)
Moderate (1%–5% normal FIX activity)	4 (19.0)	1 (16.7)	9 (19.6)	14 (19.2)
Severe (<1% normal FIX activity)	15 (71.4)	5 (83.3)	25 (54.3)	45 (61.6)
Missing	1 (4.8)	0 (0)	8 (17.4)	9 (12.3)
On‐demand treatment, *n* (%)				6 (8.2)
Prophylaxis regimen,^a^ *n* (%)				67 (91.8)
More frequent than 7 days	1 (4.8)	1 (16.7)	2 (4.3)	4 (5.5)
7‐day	17 (81.0)	5 (83.3)	32 (69.6)	54 (74.0)
10‐day	1 (4.8)	0	5 (10.9)	6 (8.2)
14‐day	0	0	5 (10.9)	5 (6.8)
21‐day	NA	NA	1 (2.2)	1 (1.4)
Other	2 (9.5)	0	1 (2.2)	3 (4.1)^b^
Other factor concentrates in the past 12 months (except rIX‐FP),^c^ *n* (%)				
rFIX (nonacog alfa, BeneFIX, Pfizer)	2 (40.0)		4 (25.0)	6 (28.6)
rFIX (coagulation FIX, Berinin, CSL Behring)	0 (0.0)		6 (37.5)	6 (28.6)
pdFIX (human plasma‐derived coagulation FIX, Haemonine, Biotest)	1 (20.0)		3 (18.8)	4 (19.0)
rFIX (human coagulation FIX, Octanine, Octapharma)	1 (20.0)		1 (6.3)	2 (9.5)
rFIXFc (eftrenonacog alfa, ALPROLIX, Sanofi)	0 (0.0)	NA	1 (6.3)	1 (4.8)
pdFIX (human plasma‐derived coagulation FIX, MONONINE, CSL Behring)	0 (0.0)		1 (6.3)	1 (4.8)
Missing	1 (20.0)		0 (0.0)	1 (4.8)

Abbreviations: FIX, factor IX; *N*, total number of patients analysed per group; *n*, number of patients; NA, not applicable; pdFIX, plasma‑derived factor IX; rFIX, recombinant factor IX; SD, standard deviation.

^a^ Patients who switched dosing frequency for prophylaxis will appear in multiple categories (regimen switched from a 3‐day to a 4‐day to a 5‐day regimen [*n* = 1]; regimen switched from a twice‐weekly to three‐times weekly regimen [*n* = 1]; regimen switched from a 7‐day to 10‐day regimen [*n* = 1]; regimen switched from a 7‐day to 14‐day regimen [*n* = 1]; regimen switched from a 10‐day to 14‐day regimen [*n* = 1]; regimen switched from a 14‐day to 21‐day regimen [*n* = 1]; regimen switched from a 14‐day to 7‐day regimen [*n* = 1]; regimen switched from a 10‐day to 7‐day regimen [*n* = 1]; regimen switched from an 8‐day to 9‐day regimen [*n* = 1]).

^b^ 'Other’ included one patient on an 8‐day regimen which was later increased to a 9‐day regimen, one patient on a 9‐day regimen and one patient on an 11‐day regimen.

^c^ Multiple answers were possible.

The majority of patients were initiated on a prophylaxis regimen with rIX‐FP (91.8%, *n* = 67/73; Table 1). The remaining 8.2% (*n* = 6/73) were initiated on an on‐demand regimen. The most common prophylaxis regimen was a 7‐day interval (74.0%, *n* = 54), followed by a 10‐day (8.2%, *n* = 6) and then a 14‐day (6.8%, *n* = 5) regimen. One patient had a 21‐day prophylaxis dosing regimen, four patients had a dosing regimen that was more frequent than every 7 days and adjusted as required (one patient received prophylaxis every 3–5 days, one twice a week, one two or three times a week, and one three times a week), and three patients had other regimens. Patients who switched dosing frequency for prophylaxis were counted in multiple categories. The most frequent weekly prophylaxis doses for children and adolescents were 35–50 IU/kg (children, 36.8%; adolescents, 60.0%); the most frequent weekly dose for adults was 25–<35 IU/kg (36.6%).

### 3.2 Bleeding Rates

Bleeding rates are summarised in Figure 2. For those treated with any prophylaxis regimen, the median (range) ABR was 0.89 (0.0–9.6). The median ABR was 1.12, 0.68 and 0.13 for those treated with a 7‐, 10‐ or 14‐day regimen, respectively. The median ABR was 0.36 for those treated with an on‐demand regimen. The respective median (range) ABR for those children, adolescents and adults treated with any prophylaxis regimen was 2.08 (0.0–9.6), 0.86 (0.0–2.4) and 0.52 (0.0–6.5).

FIGURE 2Summary of (a) ABRs and (b) AsBRs^a^. ^a^Patients without any time on prophylaxis/on demand were excluded. ^b^Any regimen included the ‘other prophylaxis’ regimen. ^c^Patients who did not fill out diaries or had <12 weeks on a specific regimen were set to missing in the ABR for that regimen. Only bleeding episodes requiring treatment were included. ABR, annualised bleeding rate; AsBR, annualised spontaneous bleeding rate; IQR, interquartile range; max, maximum; min, minimum; *N*, total number of patients analysed per group; *n*, number of patients; NA, not applicable; SD, standard deviation.

**Figure:**
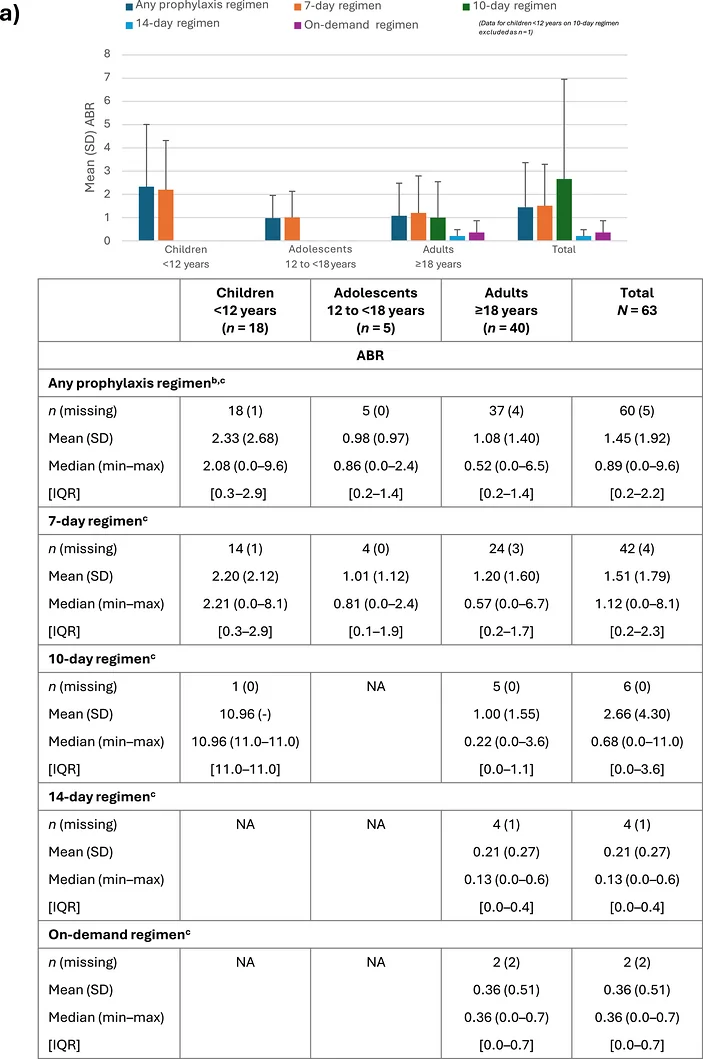

**Figure:**
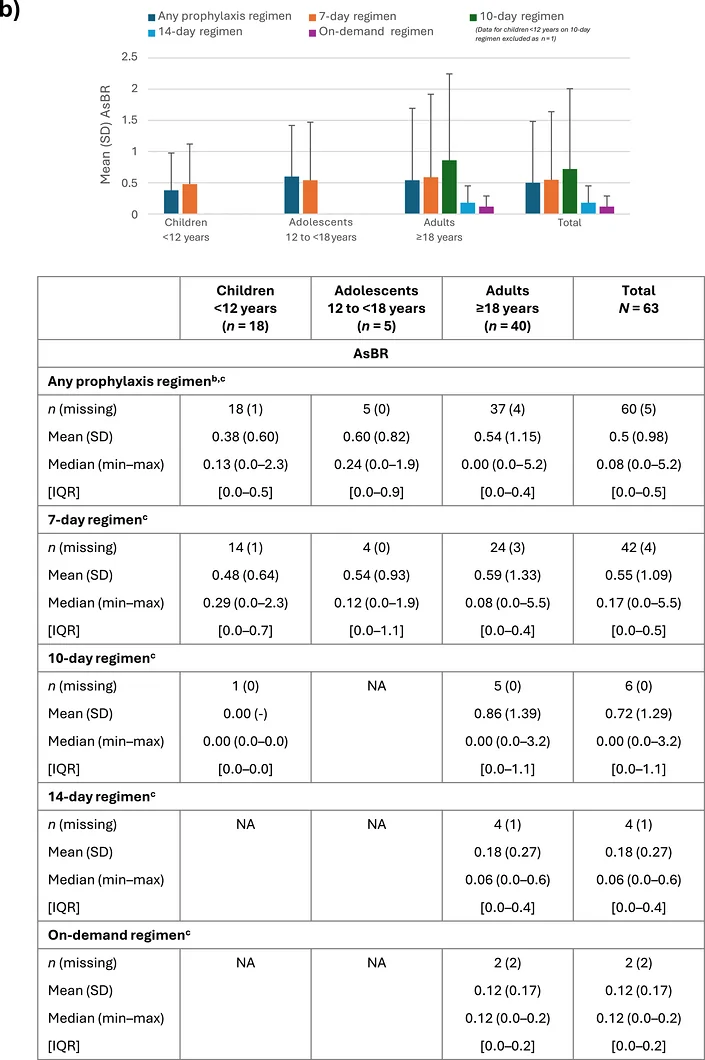

For those treated with any prophylaxis regimen, the median (range) AsBR was 0.08 (0.0–5.2). The median AsBR was 0.17, 0.00 and 0.06 for those treated with a 7‐, 10‐ or 14‐day regimen, respectively. The median AsBR was 0.12 for those treated with an on‐demand regimen. The respective median (range) AsBR for those children, adolescents and adults treated with any prophylaxis regimen was 0.13 (0.0–2.3), 0.24 (0.0–1.9) and 0.00 (0.0–5.2).

Investigators rated the overall haemostatic effectiveness as ‘good’ or ‘excellent’ in 100% (*n* = 63) of cases based on the best assessment and in 94% of cases based on the worst assessment (*n* = 63). In the prophylaxis group, there were 302 bleeds requiring treatment; the majority (77.2%) were treated with 1–2 infusions of rIX‐FP and 22.8% required >2 infusions to treat. In the on‐demand group, there were three bleeds requiring treatment; all of which were treated with 1–2 infusions of rIX‐FP.

### 3.3 FIX Consumption

Median (range) annual FIX consumption with any prophylaxis regimen was comparable between children and adolescents (2,493.6 [835–4,857] IU/kg and 2,471.7 [1,931–4,557] IU/kg, respectively) and was lower for adults (1,808.6 [698–3,764] IU/kg). Median weekly rIX‐FP consumption on any prophylaxis regimen was 45.00 IU/kg for children, 43.00 IU/kg for adolescent patients and 32.50 IU/kg for adult patients in the EFS (Figure 3). Median weekly rIX‐FP consumption on any prophylaxis regimen by severity was 39.00, 28.00 and 27.50 IU/kg for patients with severe, moderate and mild haemophilia B, respectively. Median rIX‐FP dose was 37.50, 42.50, 47.00 and 65.00 IU/kg per infusion for patients on 7‐, 10‐, 14‐ and 21‐day prophylaxis treatment regimens, respectively (Figure 4).

FIGURE 3Summary of rIX‐FP consumption by haemophilia B severity. ^a^Effectiveness data set, *N*  =  59. ^b^Frequency and dosage of infusions for prophylaxis were calculated based on 100% compliance of the prescribed regimen. ^c^Patients who switched dosing frequency for prophylaxis appear multiple times. max, maximum; min, minimum; *N*, total number of patients analysed per group; *n*, number of patients; NA, not applicable; SD, standard deviation.

**Figure:**
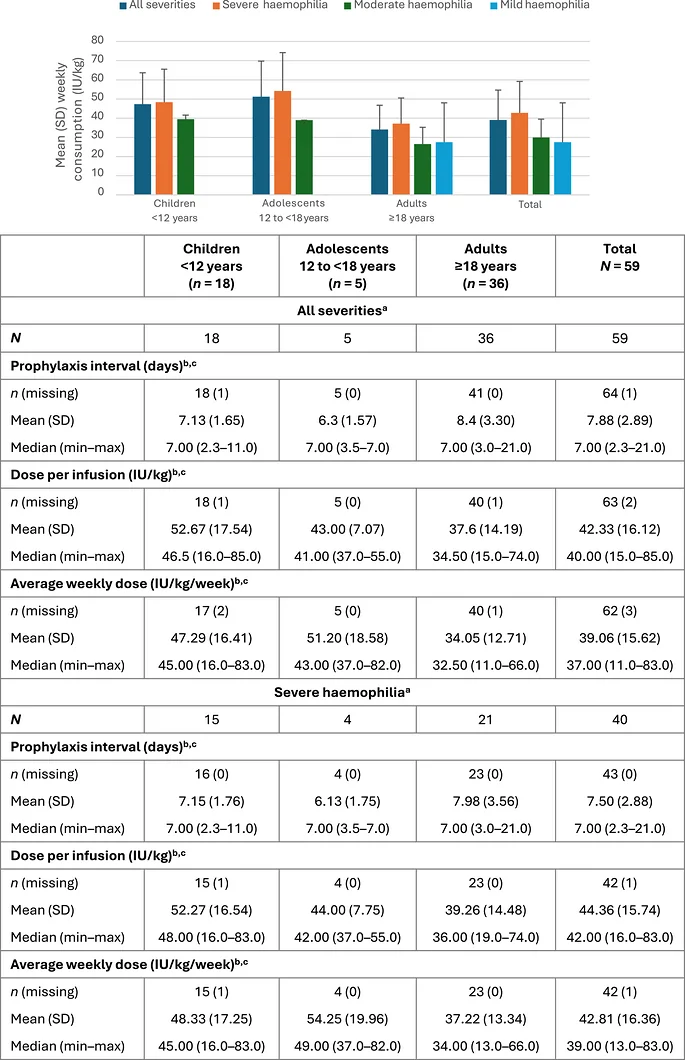

**Figure:**
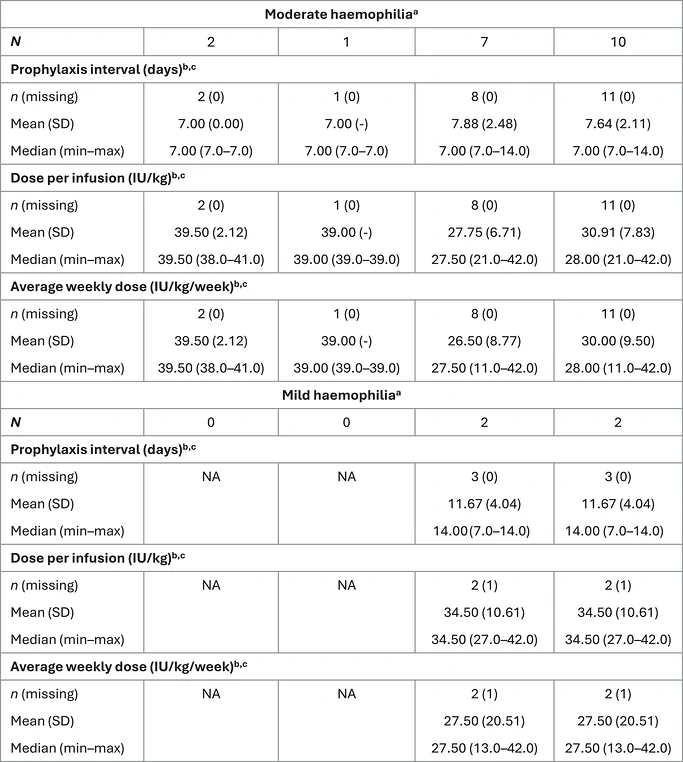

**FIGURE 4:**
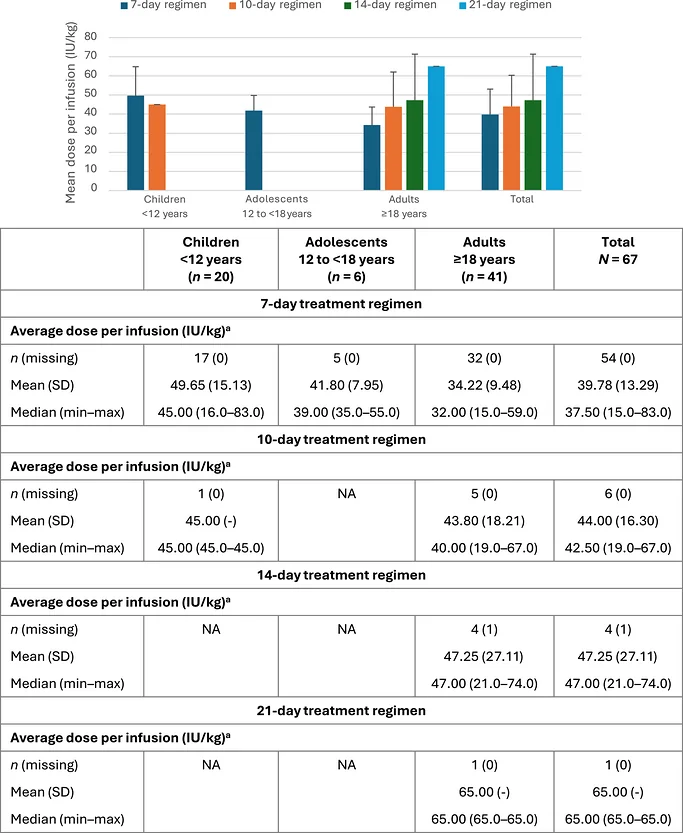
Summary of rIX‐FP dose by regimen. ^a^Average dose per infusion was rounded to integer values. max, maximum; min, minimum; *N*, total number of patients analysed per group; *n*, number of patients; NA, not applicable; SD, standard deviation.

### 3.4 Safety Analysis

In total, 82 AEs in 34 patients were reported (Table 2), none of which were considered related to rIX‐FP. Most AEs were not serious. Throughout the study, 19 SAEs in 10 patients were reported. There were four patients who had a serious bleeding event. The most frequent AEs were fall (8.2%, *n* = 6), arthralgia and haematoma (both 5.5 %, *n* = 4). No patient developed FIX inhibitors, and no patient deaths were reported.

**TABLE 2:** Incidence of AEs.

	Full analysis set *N* = 73		
	Number of events	Number of patients with an event	
	*n*	*n*	%
AEs	82	34	46.6
AEs classified as FIX inhibitor formation	0	0	0.0
SAEs	19	10	13.7
Adverse drug reactions	0	0	0.0
Serious adverse drug reactions	0	0	0.0
AEs leading to death	0	0	0.0

Incidence of AEs by pre‐specified category, preferred term and seriousness			
	Non‐serious *n* (%)	Serious *n* (%)	All *n* (%)
Any AE	29 (39.7)	10 (13.7)	34 (46.6)
FIX inhibitor formation during therapy with rIX‐FP	0 (0.0)	0 (0.0)	0 (0.0)
Serious bleeding	0 (0.0)	4 (5.5)	4 (5.5)
Cerebral haematoma	0 (0.0)	1 (1.4)	1 (1.4)
Cerebral haemorrhage	0 (0.0)	1 (1.4)	1 (1.4)
Craniocerebral injury	0 (0.0)	1 (1.4)	1 (1.4)
Gastric ulcer haemorrhage	0 (0.0)	1 (1.4)	1 (1.4)
Hiatus hernia	0 (0.0)	1 (1.4)	1 (1.4)
Mucous membrane disorder	0 (0.0)	1 (1.4)	1 (1.4)
Oesophageal varices haemorrhage	0 (0.0)	1 (1.4)	1 (1.4)
Lack of drug effect	0 (0.0)	0 (0.0)	0 (0.0)
Pharmacological interaction between two drugs	0 (0.0)	0 (0.0)	0 (0.0)

Abbreviations: AE, adverse event; FIX, factor IX; *N*, total number of patients analysed per group; *n*, number of patients; SAE, serious adverse event.

### 3.5 Quality of Life, Patient Satisfaction and Absences

Mean (SD) Haemo‐QoL and Haem‐A‐QoL results indicated a comparable good quality of life at study start (total score Haemo QoL II 20.0 [12.95]; Haemo QoL III 19.6 [9.40] and Haem A QoL 22.3 [14.18]). A low number of completed paediatric Haemo‐QoL questionnaires were received (ages 4 to 7: *n* = 0/10; ages 8 to 12: *n* = 4/7; ages 13 to 18: *n* = 4/7) and no consistent trend was observed across different subscales of the questionnaires at follow‐up compared with initial assessment. Due to the low number of completed questionnaires (Table 3), and the comparatively large amount of missing data, results should be interpreted with caution.

**TABLE 3:** Haem‐A‐QoL results in adults (≥16 years).

	Haem‐A‐QoL Total (*N* = 40)^a^		
	Initial assessment	Follow‐up assessment^b^	Within‐patient difference
Standardised Total Score (%)^c^			
*n* (missing)	37 (3)	36 (4)	34 (6)
Mean (SD)	22.3 (14.18)	23.2 (15.51)	0.5 (9.45)
Median (min–max)	21.1 (4–58)	19.3 (5–64)	0.4 (−18–19)
Standardised Physical Health Score (%)^c^			
*n* (missing)	37 (3)	36 (4)	34 (6)
Mean (SD)	23.1 (24.62)	28.6 (25.59)	3.7 (18.07)
Median (min–max)	10.0 (0–70)	25.0 (0–90)	0.0 (−35–45)
Standardised Feeling Score (%)^c^			
*n* (missing)	37 (3)	36 (4)	34 (6)
Mean (SD)	18.1 (19.86)	17.0 (19.28)	−1.7 (−17.56)
Median (min–max)	12.5 (0–75)	9.4 (0–75)	0.0 (−50–50)
Standardised View of Yourself Score (%)^c^			
*n* (missing)	38 (2)	36 (4)	35 (5)
Mean (SD)	27.1 (19.82)	26.0 (19.00)	−11.0 (17.02)
Median (min–max)	25.0 (0–80)	22.5 (0–75)	0.0 (−35–45)
Standardised Family Planning Score (%)^c^			
*n* (missing)	26 (14)	24 (16)	20 (20)
Mean (SD)	10.1 (23.72)	6.3 (14.63)	1.3 (7.20)
Median (min–max)	0.0 (0–100)	0.0 (0–50)	0.0 (−13–25)
Standardised Sports and Leisure Score (%)^c^			
*n* (missing)	34 (6)	32 (8)	30 (10)
Mean (SD)	43.7 (28.09)	39.4 (30.90)	−2.4 (16.24)
Median (min–max)	50.0 (0–95)	37.5 (0–100)	−5.0 (−40–29)
Standardised Work and School Score (%)^c^			
*n* (missing)	34 (6)	33 (7)	31 (9)
Mean (SD)	10.7 (17.10)	11.2 (21.12)	−1.2 (13.64)
Median (min–max)	0.0 (0–69)	0.0 (0–88)	0.0 (−31–31)
Standardised Dealing with Haemophilia Score (%)^c^			
*n* (missing)	37 (3)	36 (4)	34 (6)
Mean (SD)	20.0 (24.01)	20.8 (22.23)	2.5 (28.24)
Median (min–max)	16.7 (0–100)	16.7 (0–100)	0.0 (−83–83)
Standardised Treatment Score (%)^c^			
*n* (missing)	38 (2)	36 (4)	35 (5)
Mean (SD)	24.9 (15.89)	22.6 (15.74)	−1.3 (12.85)
Median (min–max)	23.4 (0–81)	18.8 (0–59)	0.0 (−31–25)
Standardised Future Score (%)^c^			
*n* (missing)	38 (2)	36 (4)	35 (5)
Mean (SD)	30.4 (21.48)	34.2 (24.60)	4.3 (16.50)
Median (min–max)	25.0 (0–80)	30.0 (0–90)	0.0 (−35–35)
Standardised Partnership and Sexuality Score (%)^c^			
*n* (missing)	37 (3)	36 (4)	34 (6)
Mean (SD)	5.9 (13.87)	12.3 (23.10)	6.9 (18.40)
Median (min–max)	0.0 (0–67)	0.0 (0–92)	0.0 (−8–83)

Abbreviations: max, maximum; min, minimum; *N*, total number of patients analysed per group; *n*, number of patients; SD, standard deviation.

^a^ Patients who did not complete the questionnaire were displayed as missing.

^b^ If there was more than one follow‐up assessment for the questionnaire, the last one was displayed.

^c^ Higher scores indicated a higher burden of disease.

The majority of patients were ‘very satisfied’ (52.4%, *n* = 33) or ‘satisfied’ (33.3%, *n* = 21) with rIX‐FP treatment; data were missing for seven (11.1%) patients. No patients were ‘dissatisfied’ with rIX‐FP treatment. The percentage of patients who were ‘satisfied’ or ‘very satisfied’ was largely comparable throughout the age groups (children: 77.8%, *n* = 14; adolescents: 80.0%, *n* = 4; adults: 90.0%, *n* = 36).

In total, there was a very small number of haemophilia‐related absences recorded over the course of the study. Overall, the mean (SD) annual number of haemophilia‐related absences from work/school was 1.6 (9.84) days; it was highest for adults (2.4 [12.32] days) compared with children (0.3 [1.01] days) and adolescents (0.2 [0.44] days).

### 3.6 Subgroup Analysis of PUPs

There were nine PUPs with a median (min, max) age of 3.0 (1, 11) years, including two infants (<2 years of age) and seven children between 2–12 years of age (Table 4). Six (66.7%) had severe haemophilia B, one each of the remaining three had mild haemophilia B, moderate haemophilia B or missing data. Most patients (88.9%, *n* = 8) were initiated on a prophylaxis regimen, of which the majority (*n* = 6) were treated with a 7‐day regimen. The remaining patient, with mild disease severity, was initiated on an on‐demand regimen. Gene mutations identified in the PUPs included four patients (44.4%) with missense mutation, one (11.1%) with a nonsense mutation, and one (11.1%) with an intron/exon mutation; data for three patients were missing.

**TABLE 4:** Baseline characteristics of PUPs.

	PUPs (*n* = 9)
Sex, *n* (%)	
Male	9 (100)
Female	0 (0)
Age	
Mean (SD)	3.4 (3.09)
Median (range)	3.0 (1–11)
Age groups, *n* (%)	
Infants: 0 to <2 years	2 (22.2)
Children: 0 to <12 years	7 (77.8)
Weight (kg)	
Median (range)	15 (8–29)
Severity of haemophilia,^a^ *n* (%)	
Mild (>5% normal FIX activity)	1 (11.1)
Moderate (1%–5% normal FIX activity)	1 (11.1)
Severe (<1% normal FIX activity)	6 (66.7)
Missing	1 (11.1)
Gene mutation, *n* (%)	
Nonsense	1 (11.1)
Missense	4 (44.4)
Intron/exon	1 (11.1)
Missing	3 (33.3)
On‐demand treatment, *n* (%)	1 (11.1)
Prophylaxis regimen, *n* (%)	8 (88.9)

Abbreviations: FIX, factor IX; *n*, number of patients; SD, standard deviation.

^a^ Based on FIX activity at initial diagnosis.

For the seven children between 2 and 12 years of age on any prophylaxis regimen included in the EFS, the median (range) ABR was 2.16 (0.4–8.1) and the median (range) AsBR was 0.26 (0.0–0.7) (Figure 5). For children treated with a 7‐day regimen, the median AsBR was 0.35. Sixty‐seven bleeding episodes were recorded during the whole observation period in the prophylaxis regimen. In 71.2% (*n* = 47) of the bleeding episodes, only one infusion of rIX‐FP was required to treat the bleed, while 15.2% (*n* = 10) of bleeding episodes required two infusions. Two or more infusions were required for 13.6% (*n* = 9) of bleeding episodes.

**FIGURE 5:**
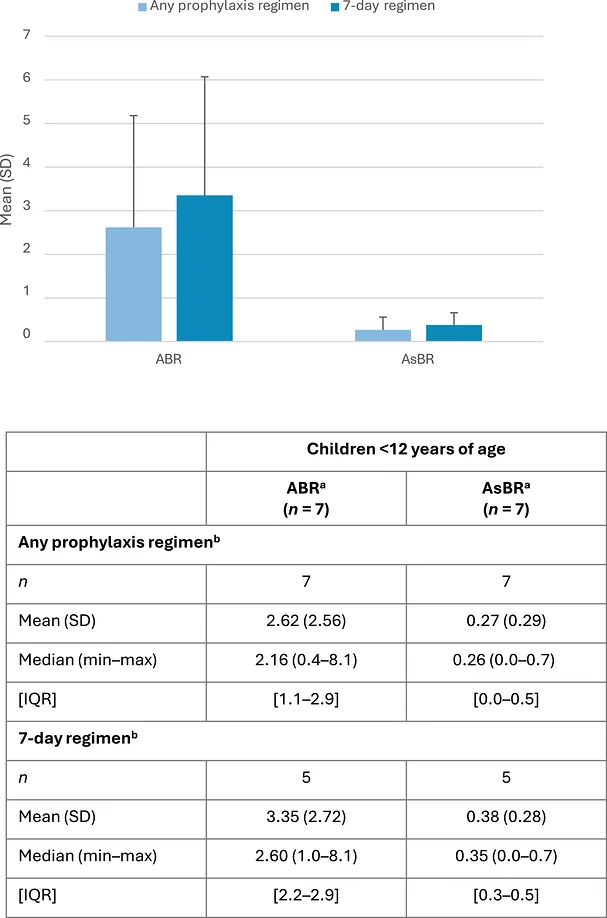
Summary of bleeding rate for PUPs treated with prophylaxis^a^. ^a^Only bleeds requiring treatment. ^b^Patients who did not fill out diaries or patients with <12 weeks on a specific regimen were set to missing in the ABR/AsBR for that regimen. ABR, annualised bleeding rate; AsBR, annualised spontaneous bleeding rate; IQR, interquartile range; max, maximum; min, minimum; *n*, number of patients; SD, standard deviation.

The median (range) average weekly dose for PUPs treated with a prophylaxis regimen was 60.5 (27.0–67.0) IU/kg and the median (range) average dose per infusion was 63.0 (56.0–85.0) IU/kg. The median (range) annual FIX consumption for the six children on prophylaxis treatment was 3,324.3 (1,451–4,857) IU/kg.

A total of 9 AEs were experienced by 3 (33.3%) PUPs; all were non‐serious. No events of FIX inhibitor formation or adverse drug reactions were reported.

## 4 Discussion

To our knowledge, this is the largest real‐world study of factor replacement therapy in PwHB in Germany to date. The results of this prospective, multicentre, non‐interventional study in Germany indicate that rIX‐FP prophylaxis is effective and well tolerated in patients of all ages in routine clinical practice. Over 2 years of treatment, rIX‐FP provided excellent bleed control in patients of all ages. The overall median ABRs for the 10‐day and 14‐day prophylaxis regimens were numerically lower than for the 7‐day regimen (0.68 and 0.13, respectively, vs 1.12). However, as expected, children had a higher median ABR than adults and adolescents (any prophylaxis regimen: ABR: 2.08 vs 0.52 and 0.86, respectively). With regards to spontaneous bleeds, the overall median AsBRs for the 10‐day and 14‐day prophylaxis regimens were numerically lower than for the 7‐day regimen (0.00 and 0.06, respectively, vs 0.17). However, the median AsBR was highest in the adolescent age group (0.24) in comparison with adults (0.00) and children (0.13).

Whilst the median ABR was numerically higher in the 7‐day versus 10‐ and 14‐day intervals, this is consistent with the Phase 3 extension study [8] and does not indicate reduced effectiveness of rIX‐FP. Median ABRs in the extension study were 1.33, 0.80 and 0.92 for those on 7‐, 10‐ and 14‐day intervals, respectively [8]horter dosing intervals may be prescribed for various reasons and patients on shorter intervals may have a higher bleeding tendency, which may explain the higher bleeding rate in the 7‐day group. As this was not part of the study assessment, findings should be interpreted with caution. Overall, rIX‐FP delivered effective bleed protection across all dosing intervals and offered patients the choice of extended intervals.

With rIX‐FP, dosing regimens can be tailored to individual patients with the option to extend dosing frequency up to 21 days per the label, which is not currently possible on any other SHL/EHL label. Most patients on prophylaxis were dosed once every 7 days, but selected adult patients were able to extend their dosing regimens to every 14 or 21 days. All patients on prophylaxis were well controlled with comparable effectiveness, regardless of the dosing regimen. A similar pattern of results was shown in patients who received on‐demand treatment with rIX‐FP. The PUPs subgroup analysis also demonstrated rIX‐FP to be similarly effective without any safety concerns or inhibitor development.

Data from routine clinical practice on the use of FIX products are important to confirm the outcomes of clinical trials and to provide data which are more representative of the whole haemophilia population [10, 11, 12, 13]. The results of this non‐interventional study align with the results of the PROLONG‐9FP clinical trial with rIX‐FP, which demonstrated the efficacy of rIX‐FP at 21‐day dosing intervals [5, 6, 7, 8, 9]. The results from this study also align with previous retrospective studies published for Germany [10], Italy [13, 14, 15], Belgium [11], France [16] and the UK [11], evaluating clinical outcomes in PwHB who switched to rIX‐FP from a previous FIX product. Furthermore, these results also align with other, ongoing prospective multicentre studies in Italy [13, 15, 17] and Spain [18]. Altogether, these studies support the use of rIX‐FP to reduce the patient treatment burden, with the option to reduce the infusion frequency whilst maintaining bleed protection with high FIX levels, along with a tolerable safety profile.

Prophylaxis is the current standard of care for patients with severe haemophilia [1]; however, this treatment is associated with a high treatment burden because of the need for repeated, lifelong, intravenous infusions. To address these limitations, other treatment options have emerged including gene therapy, which has the potential to correct the underlying mutation causing haemophilia B; however, currently gene therapy is restricted for use in eligible adults only [19, 20]. Non‐factor therapies offer subcutaneous administration often at a lower frequency than EHL FIX products to reduce treatment burden [21]; though important considerations remain, including safety signals and the optimal approach for haemostatic monitoring, and there is a need for further research [21, 22]. EHL FIX products are pivotal in the treatment of haemophilia B across all patient populations and enable a reduced treatment burden for patients because of the reduction in the number of infusions and FIX consumption, while maintaining effective bleed control. This study reinforced the effectiveness and safety of rIX‐FP across extended dosing regimens, providing the opportunity for patients to personalise their treatment to their lifestyle.

## 5 Limitations

One general limitation of real‐world studies is the small number of patients who can be recruited. However, this limitation does not apply to the current study of 73 patients. It is assumed that the patients included in this study are representative of the wider patient population; however, the results may be affected by the treatment practices of the participating centres. Due to the nature of a non‐interventional study and the limited degree of monitoring and data cleaning possible, there may be data discrepancies that cannot be completely ruled out. Additionally, there was a large amount of missing data, particularly for the quality of life questionnaires, which may have affected the interpretation of the results.

## 6 Conclusion

This non‐interventional study in Germany shows that rIX‐FP prophylaxis is effective and well tolerated in adults, adolescents and children, including PUPs, with haemophilia B. Furthermore, no other SHL/EHL allows the flexibility of individualised patient dosing with the option to extend dosing frequency up to 21 days on label. This non‐interventional study contributes to a broader knowledge of rIX‐FP in a real‐world setting and reinforces the results of the clinical trials.

## Author Contributions

All authors contributed to the design of the study, acquisition and interpretation of data and critically reviewed the manuscript. All authors approved the final version of the manuscript and agree to be accountable for all aspects of the work in ensuring that questions related to the accuracy or integrity of any part of the work are appropriately investigated and resolved.

## Funding

This study was funded by CSL Behring.

## Ethics Approval

This non‐interventional study was approved by an independent ethics committee and conducted in accordance with the requirements and ethical principles set forth in the Declaration of Helsinki, and followed local guidelines for observational studies in Germany using human data.

## Patient consent

Written and informed consent was provided by adult patients, or a parent/legal guardian in the case of minors.

## Conflicts of Interest

Johannes Oldenburg received research funding from Bayer, Biotest, CSL Behring, Octapharma, Pfizer, Swedish Orphan Biovitrum and Takeda; received consultancy, speakers bureau, honoraria, scientific advisory board and travel expenses from Bayer, Biogen Idec, BioMarin, Biotest, Chugai, CSL Behring, Freeline, Grifols, LFB, Novo Nordisk, Octapharma, Pfizer, Roche, Sanofi, Spark Therapeutics, Swedish Orphan Biovitrum and Takeda. Swee Wenning received study support, consultancy, speaker fees and travel expenses from Bayer, Biotest, CSL Behring, Novo Nordisk, Octapharma, Pfizer, Roche, Swedish Orphan Biovitrum and Takeda. Susanne Holzhauer received grants/research support from Pfizer and Swedish Orphan Biovitrum; received consultancy and speaker fees from Bayer, Pfizer, Roche, BioMarin and Swedish Orphan Biovitrum. Wolfgang Miesbach received grant and research support from Bayer, Biotest, CSL Behring, LFB, Novo Nordisk, Octapharma, Pfizer, Swedish Orphan Biovitrum, Takeda and UniQure; received consultation fees from BioMarin, Freeline, LFB, Octapharma, Swedish Orphan Biovitrum, Novartis, Novo Nordisk, Octapharma, Pfizer and Roche; and received speaker bureau fees from Shire, Pfizer, Bayer, Swedish Orphan Biovitrum, Amgen, Novartis, Alexion, Leo Pharma, Grifols, Roche and Novo Nordisk. Robert Klamroth received grants and research support from Bayer, Novo Nordisk and Swedish Orphan Biovitrum; received honoraria and consultation fees from Bayer, BioMarin, CSL Behring, Novo Nordisk, Octapharma, Pfizer, Roche, Sobi and Takeda; participates in a company‐sponsored speakers bureau for Bayer, BioMarin, CSL Behring, Novo Nordisk, Octapharma, Pfizer, Roche, Swedish Orphan Biovitrum and Takeda. Thomas Linhoff and Patrick Sommerer are CSL Behring employees. Christian Pfrepper received speaker honoraria from BMS, CSL Behring, Pfizer, Roche and Shire Takeda; acted as a medical advisor for Bayer HealthCare, Chugai, CSL Behring, Novo Nordisk, Pfizer, Roche and Shire Takeda; received research grants from Fujimori, Leo Pharma, Takeda and Roche. Martin Olivieri received grants/research support from Bayer, Biotest, Takeda, CSL Behring, Octapharma, Pfizer, Shire, Roche and Swedish Orphan Biovitrum; received consultancy and speaker fees from Bayer, Biotest, Novo Nordisk, Takeda, CSL Behring, Pfizer, Roche, Stago and Swedish Orphan Biovitrum.

## Data Availability

The study has been registered in the non‐interventional studies registry at the Paul Ehrlich Institute (German federal authority for vaccines and biomedicines; study code CSL654_5005; NIS number 410). Please visit https://www.pei.de/SharedDocs/awb/nis‐0401‐0500/0410.html for more information and to view the clinical study report. The data from this study have not been published in a repository; contact MedWiss@cslbehring.com for additional information.
